# Enhancing land cover object classification in hyperspectral imagery through an efficient spectral-spatial feature learning approach

**DOI:** 10.1371/journal.pone.0313473

**Published:** 2024-12-05

**Authors:** Masud Ibn Afjal, Md. Nazrul Islam Mondal, Md. Al Mamun

**Affiliations:** 1 Department of Computer Science and Engineering, Hajee Mohammad Danesh Science and Technology University, Dinajpur, Bangladesh; 2 Department of Computer Science and Engineering, Rajshahi University of Engineering & Technology, Rajshahi, Bangladesh; Xidian University, CHINA

## Abstract

The classification of land cover objects in hyperspectral imagery (HSI) has significantly advanced due to the development of convolutional neural networks (CNNs). However, challenges such as limited training data and high dimensionality negatively impact classification performance. Traditional CNN-based methods predominantly utilize 2D CNNs for feature extraction, which inadequately exploit the inter-band correlations in HSIs. While 3D CNNs can capture joint spectral-spatial information, they often encounter issues related to network depth and complexity. To address these issues, we propose an innovative land cover object classification approach in HSIs that integrates segmented principal component analysis (Seg-PCA) with hybrid 3D-2D CNNs. Our approach leverages Seg-PCA for effective feature extraction and employs the minimum-redundancy maximum relevance (mRMR) criterion for feature selection. By combining the strengths of both 3D and 2D CNNs, our method efficiently extracts spectral-spatial features. These features are then processed through fully connected dense layers and a softmax layer for classification. Extensive experiments on three widely used HSI datasets demonstrate that our method consistently outperforms existing state-of-the-art techniques in classification performance. These results highlight the efficacy of our approach and its potential to significantly enhance the classification of land cover objects in hyperspectral imagery.

## 1 Introduction

Land cover object classification performs a crucial role in numerous fields, including agriculture, urban planning, environmental monitoring, and resource management [1–3]. Hyperspectral images (HSIs) have significantly advanced this classification task due to their ability to provide detailed spectral information for each pixel. These images offer both 2D spatial information and 1D spectral information, providing rich data that poses significant analytical challenges due to its high dimensionality. HSIs also have diverse applications in multimedia, medicine, atmospheric monitoring, target detection, hydrological assessment, segmentation, anomaly detection, and wind forecasting [4–8]. Traditional classification methods, such as Support Vector Machines (SVM) [9], K-Nearest Neighbor (KNN) [10], and Artificial Neural Networks (ANN) [11], primarily rely on spectral information and utilize feature extraction and dimensionality reduction techniques like Principal Component Analysis (PCA) [12, 13], Linear Discriminant Analysis (LDA) [14, 15], Independent Component Analysis (ICA) [16], and Minimum Noise Fraction (MNF) [17].

PCA, for example, identifies features by capturing variations and transforming them into principal components (PCs). LDA increases class separation by extracting relevant features, while MNF arranges data based on the signal-to-noise ratio (SNR) axis. However, the high data dimensionality present in HSIs can lead to the Hughes phenomenon, where classification accuracy initially rises and then declines [18]. To mitigate this, dimensionality reduction techniques like PCA and LDA have been employed, but they can introduce noise.

Feature selection is a critical aspect of machine learning preprocessing, involving the identification of relevant features while eliminating irrelevant or redundant ones from the initial feature subset. Common feature selection strategies include Mutual Information (MI) [19], Normalized Mutual Information (nMI) [20], and Minimum Redundancy-Maximum Relevance (mRMR) [21]. However, a significant challenge with hyperspectral images is the lack of spatial characteristics, limiting their classification using popular machine learning methods like KNN and SVM. Despite advancements in hyperspectral image classification, traditional methods often neglect spatial context, relying solely on spectral information. This oversight results in suboptimal performance, highlighting the need for novel approaches that integrate spectral-spatial features for improved land cover object identification.

In computer vision, Deep learning (DL) has emerged as a dominant approach due to its ability to automatically extract complex features. DL has been applied to HSI classification, with various architectures and techniques proposed [22–24]. However, DL methods for HSI classification primarily prioritize spectral information, often overlooking spatial context, which can limit their performance. In response, researchers have employed 2D convolutional neural networks (CNNs) [25–27], which capture spatial patterns and inspire innovative architectures. Other approaches include pixel-pair methods that exploit the similarity between neighboring pixels and small-scale data-driven methods for limited samples [28, 29]. Efforts to enhance HSI classification by combining spectral and spatial features [30–32] have shown that fusion of these two types of information enables algorithms to leverage both spectral diversity and spatial context, achieved through network transformations or double-branch spatial-spectral extraction and fusion.

CNNs have strong feature extraction capabilities and have been enhanced to address their limitations [33]. For example, in random weight network [34], dense residual networks where the skip connections ability facilitates smooth gradient flow [35] and have been applied to HSI classification. Deep residual networks with attention mechanisms [36] have also shown promising results. Additionally, GPU development has reduced training time for large parameter networks [37]. Hybrid models that combine 2D-CNN and 3D-CNN layers have been proposed to utilize both spectral and spatial feature maps for maximum accuracy [38–40]. These models address the limitations of using either 2D [25, 26] or 3D CNNs [41, 42], as they may not effectively extract discriminating feature maps from the spectral dimension alone or become computationally complex for classes that have the same texture patterns towards various spectral bands.

Recent advancements have introduced hybrid models that extend CNN capabilities. Hybrid-2DNET [43] enhances SpectralNet by integrating feature selection post-factor analysis, identifying high-impact features for improved classification. Similarly, TP-Net [44], a triple-path spectral-spatial network, uses interleaved attention mechanisms to discern important features better. The Deep Spectral Spatial Feature Enhancement (DS2FE) [45] employs a vision-based transformer with a multiscale feature extractor for low-level spectral-spatial features and a regional attention mechanism with a spatially gated module for high-level semantic extraction. S2PNet [46], an interactive learning model, uses multi-stage spectral purification, global-local feature interaction, and shallow-deep integration to enhance classification accuracy by reducing spectral and spatial heterogeneity.

Traditional PCA, as a preprocessing step of CNN, seems a single overall model for the complete HSI [38, 47], which may not effectively capture the intricate spatial and spectral variations present in the data. However, segmented PCA [15, 48–51] offers a solution by dividing the data into smaller sub-groups and applying PCA independently to each local sub-group. This localized and adaptive approach leads to improved preservation of spatial-spectral information, consequently enhancing the accuracy of subsequent classification or analysis tasks. This research presents a hybrid spectral-spatial feature extraction technique for effective HSI classification. We first recover spectral properties in local and global domains using segmented PCA. Then, we employ the minimum-redundancy maximum-relevance (mRMR) technique to remove additional duplicate information and select more accurate and meaningful features from the extracted PCs. Subsequently, we utilize a hybrid neural network that incorporates both 3D-CNN and 2D-CNN layers to extract both spectral and spatial features from the selected PCs.

The key contributions of this paper are summarized as follows:

- An effective combination of unsupervised segmented PCA (Seg-PCA)-based feature extraction and supervised mRMR-based feature selection is presented as a preprocessing step for hybrid 3D-2D CNN.
- A novel approach is introduced that combines Seg-PCA-mRMR with hybrid spectral-spatial feature extraction using 3D-CNN and 2D-CNN combined. This enables the extraction of enhanced spectral-spatial features from HSIs for enhanced classification.
- The proposed approach incorporates network inputs and selects features from different sub-regions, leading to enhanced classification accuracy. The approach captures a more complete representation of the HSI data by extracting features from both the spectral and spatial domains.
- We conducted empirical experiments on three widely used HSI datasets, comparing our proposed framework with state-of-the-art approaches. Our method demonstrated superior performance in terms of classification accuracy, generalization, and robustness.

The remaining parts of this article are organized as follows: In-depth discussion on the theoretical basis related to the topic is presented in Section 2. Section 3 outlines the proposed neural network approach and its specific implementation steps. In Section 4, we thoroughly discuss the experimental results obtained and analyze them in detail. Lastly, Section 5 summarizes the conclusions drawn from this study.

## 2 Related methodology

### 2.1 PCA-based feature extraction for HSI

PCA is a popular feature extraction and dimensionality reduction technique used for analyzing HSI data. Its objective is to retain crucial information while transforming the high-dimensional data into a lower-dimensional space. PCA achieves this by identifying principal components (PCs) that capture maximum variance in the data [52]. To implement PCA, each sample pixel’s spectral vector in the HSI data matrix is denoted as **x**_*n*_ = [*x*_*n*1_*x*_*n*2_…*x*_*nF*_]^*T*^, with *n* ∈ [1, *S*], and *S* = *X* × *Y* representing the spatial dimensions. The data matrix **D** is used to derive the zero-mean image **I**, where **I**_*n*_ = **x**_*n*_ − **M**, and $M=\frac{1}{S}\sum_{n=1}^{S}\;x_{n}$. The covariance matrix $C=\frac{1}{S}{II}^{T}$ is computed using eigen decomposition **C** = **V** **E** **V**^**T**^, where *E* and *V* are eigenvalues and eigenvectors (PCs), respectively. A subset of *q* PCs, represented by matrix **W**, is selected based on descending eigenvalue rankings. Finally, the projection matrix **Z** of **D** is obtained as **Z** = **W**^*T*^ × **I**.

Seg-PCA implementation involves dividing the HSI data matrix into *L* subgroup datasets based on band correlations. Each subgroup captures local characteristics effectively by grouping strongly correlated bands together. Seg-PCA selectively reduces the number of spectral bands while preserving spatial dimensions. In Seg-PCA, the HSI data matrix **D** is divided into subgroups **D**_*t*_, where *t* ∈ [1, 2, ..*L*]. Eigendecomposition is applied to each **D**_*t*_ to compute covariance matrices. The projection matrices of each subgroup are then combined to obtain the overall projection matrix.

### 2.2 mRMR based feature selection for HSI

mRMR is a widely used feature selection method in the analysis of HSI data. It aims to select the most relevant features related to the target variable while minimizing redundancy among them. The process involves calculating the Mutual Information (MI) between the target variable and each feature. The features are then sorted in descending order based on their MI scores, and they are added one by one to the subset until the desired number is achieved [20, 53]. To perform the MI-based feature selection, two input images, denoted as **X** and **C**, are required, along with their respective marginal probability distributions, represented as *p*(*x*) and *p*(*c*), as well as their joint probability distribution *p*(*x*, *c*). The MI, denoted as *I*(*X*, *c*), is calculated using these probability distributions in the following equation:

$$\begin{array}{c} I\left(X,C\right)=\sum_{c=1}^{S}\sum_{x=1}^{S}p\left(x,c\right)log\frac{p(x,c)}{p(x)p(c)} \end{array}$$ (1)

In the context of HSI analysis, MI-based feature selection uses **X** as the input extracted spectral features and **C** as the corresponding ground truth image. The aim is to maximize the MI when **X** and **C** are equivalent. To avoid redundancy, the greedy approach of MI-based feature selection selects the (*k* + 1)^*th*^ feature while taking into account the previously selected *Q* features, using the following equation:

$$\begin{array}{c} G\left(X_{i},k\right)=I\left(Xi,C\right)-\beta \sum q_{j}\epsilon Q\tilde{I}\left(X_{i},q_{j}\right),X_{i}⊄Q \end{array}$$ (2)

Here, *β* regulates redundancy’s significance in the mRMR criterion, and $\tilde{I}$ is the normalized Mutual Information defined as:

$$\begin{array}{c} \tilde{I}\left(X,C\right)=\frac{I(X,C)}{\sqrt{\left(H(X)H(C)\right)}} \end{array}$$ (3)

To ensure adequate feature selection features with $\tilde{I}\left(X,C\right)<\xi$ are eliminated, where *ξ* is a threshold representing the minimum level of relevance required for the target data. Also, the condition $\tilde{G}\left(X_{i},k\right)>0$ is used to verify if the chosen feature is unalike to the already selected ones, ensuring desirable selections.

### 2.3 Convolutional Neural Network (CNN)

Indeed, CNNs are a feedforward neural network that performs remarkably in large-scale image processing tasks. CNNs are specifically designed to handle image data efficiently, using their ability to learn hierarchical features from raw pixel values automatically [54–60]. It leverages the 2D image structure, recognizing pixel correlations in adjacent regions, and utilizes feature sharing through convolution. Thus, it detects local patterns and features in the input images, making them highly effective for tasks such as image classification, object detection, and image segmentation. Due to their ability to handle large-scale image data effectively, CNNs have become the backbone of numerous remotely sensed image applications and have significantly advanced the field of spectral image processing. The CNN architecture, comprising convolutional layers, activation functions (such as ReLU), pooling layers for dimensionality reduction, and fully connected layers, has revolutionized image processing tasks. Convolutional layers apply filters to capture local patterns in the input data, while activation functions introduce nonlinearity for better learning. Pooling layers reduce data size, preventing overfitting. Fully connected layers transmit features to the classifier for making predictions. The backpropagation algorithm optimizes the network’s parameters during training, leading to improved performance and accurate image processing results. CNN-based HSI classification methods encompass 2D and 3D CNNs. For instance, Fast and Compact 3D CNN utilize iPCA and 3D-2D CNNs for feature reduction and classification [41]. SpectralNET leverages factor analysis for preprocessing to reduce data dimension and a wavelet-based architecture for spatial-spectral feature extraction [39]. Similarly, HybridSN incorporates PCA for feature reduction, three 3D CNNs, and a 2D CNN for classification [38]. These represent the foundational, cutting-edge CNN models for HSI classification. However, the field has seen recent advancements in the form of fusion or hybrid models that build upon these aforementioned CNN-based frameworks. One such example is the Hybrid-2DNET [43], which introduces novel modifications to the SpectralNet architecture. By incorporating a multi-resolution mutual information-based feature selection process subsequent to the factor analysis stage, this model enhances classification accuracy. Similarly, there is the triple-path spectral-spatial network, TP-Net [44], which employs an innovative approach to elevate classification performance. TP-Net integrates two distinct interleaved attention mechanisms into its architecture. This strategic incorporation of attention mechanisms contributes to heightened discriminative capabilities, ultimately resulting in improved classification accuracy. These approaches address the challenges of limited training samples and high dimensionality in HSI data, offering effective solutions for accurate HSI classification.

## 3 Proposed spectral-spatial feature learning approach

### 3.1 Overview

**Fig 1:**
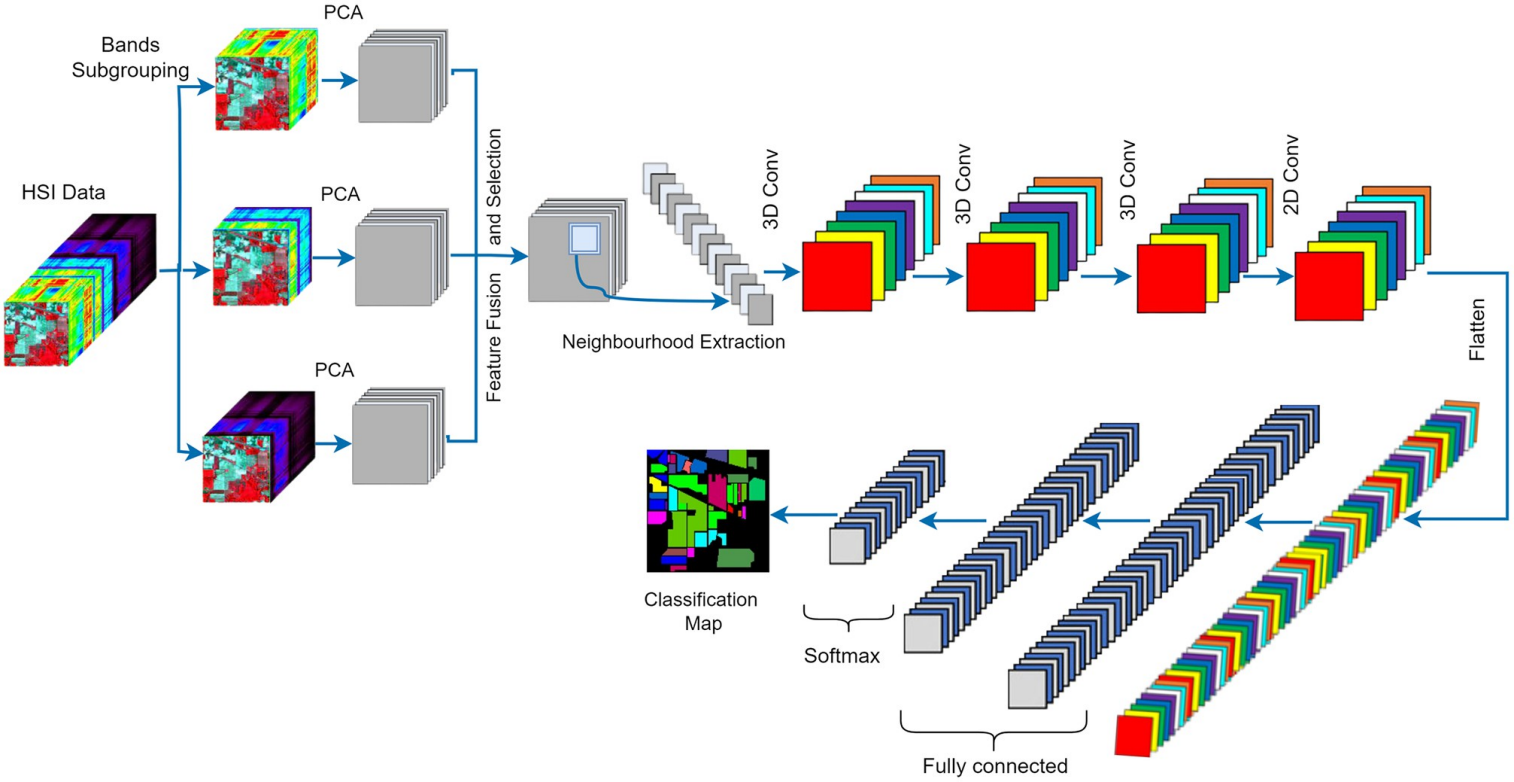
Proposed spectral-spatial feature learning approach for HSI classification.

The proposed approach for land cover object identification in HSIs, as illustrated in Fig 1, combines Seg-PCA with mRMR for feature reduction and employs a hybrid 3D-2D CNN architecture for effective spectral-spatial feature extraction. Initially, Seg-PCA is applied to reduce the dimensionality of the hyperspectral data and preserve critical spectral characteristics. Following this, mRMR is used to select the most informative features from the reduced data. The selected features are then processed through a 3D-2D CNN, which captures both spectral and spatial features for improved land cover classification. This network architecture integrates 3D convolutions to extract spectral features and 2D convolutions for spatial features, culminating in a classification layer with dense connections and dropout for robustness. By leveraging this combination of advanced techniques, the proposed method aims to significantly enhance land cover object classification performance in hyperspectral imagery.

### 3.2 Dataset description

In our experiments, we used three publicly available HSI datasets: Indian Pines (IP), University of Pavia (PU), and Salinas Scene (SA). The IP dataset contains images with a spatial dimension of 145 × 145 pixels and 224 spectral bands in the wavelength range of 400 to 2500 nm. We excluded 24 spectral bands corresponding to water absorption regions. The ground truth of the IP dataset provides labels for 16 land cover vegetation object classes [61]. The PU dataset consists of images with a spatial dimension of 610 × 340 pixels and 103 spectral bands in the wavelength range of 430 to 860 nm. The ground truth of the PU dataset classifies nine urban land cover object classes [62]. The SA dataset includes images with a spatial dimension of 512 × 217 pixels and 224 spectral bands in the wavelength range of 360 to 2500 nm. We removed 20 spectral bands affected by water absorption. The SA dataset consists of 16 different land cover object classes [63]. We selected these datasets for their diversity in spatial dimensions, spectral bands, and land-cover classes. This diversity enables us to thoroughly evaluate the performance and generalization capability of our proposed method across various HSI scenarios.

### 3.3 Seg-PCA-mRMR

While PCA is effective, it may overlook subtle information valuable for accurate HSI data analysis, particularly when the original HSI bands exhibit high correlation, resulting in a large covariance matrix and higher computational costs. To address this limitation, Seg-PCA has been proposed [48, 50] and presented [21, 49, 64, 65]. Seg-PCA is specifically designed for handling highly correlated blocks with low inter-correlations.

In Seg-PCA implementation, the HSI data matrix is divided into *L* subgroup datasets (*L* = 3 in this case) based on band correlations. Within each subgroup, strongly correlated bands are grouped together to capture local characteristics effectively. The sub-grouped data matrices are denoted as **D**_*t*_, where *t* ∈ [1, 2, 3], and each **D**_*t*_ contains *n*_*t*_ consecutive bands. Eigendecomposition is applied to each **D**_*t*_ to compute covariance matrices. The overall projection matrix is obtained by sequentially combining the projection matrices of each subgroup’s **D**_*t*_.

After applying Seg-PCA and feature extraction to the HSI dataset, we employ the mRMR feature selection process to enhance the feature set by identifying the most informative features. In the mRMR steps, we select the top *b* ranked PCs from the overall projected data, similar to our previous research with traditional machine learning [15]. The Seg-PCA-mRMR technique specifically reduces the number of spectral bands from *F* to *Q*, representing the extracted and selected PC dimensions for the reduced data. However, the spatial dimensions of the data matrix remain unchanged, with *S* = *X* × *Y*, where **D** = *X* × *Y* × *F* representing the overall input data cube. The modified input data after Seg-PCA-mRMR is represented by **Z**_*Q*_ = *X* × *Y* × *Q*, where **Z**_*Q*_ corresponds to the modified input data cube after applying Seg-PCA-mRMR. This additional step offers several advantages, such as improved computational efficiency, enhanced classification accuracy, and a deeper understanding of the underlying scientific insights present in the data. The Seg-PCA+mRMR algorithm (Algorithm 1 and 2) is employed as a preprocessing step in the proposed methodology for dimensionality reduction and feature selection. It divides the original hyperspectral data cube **D** into *L* subgroup datasets using Seg-PCA and selects the most informative principal components using mRMR.

**Algorithm 1** Seg-PCA followed by mRMR Feature Selection

**Require:** HSI data cube **D** with dimensions *X* × *Y* × *F*

1: Divide **D** into *L* subgroup datasets based on band correlations

2: **for** *t* = 1 to *L* **do**

3:  Compute covariance matrix **C**_*t*_ for **D**_*t*_

4:  Perform eigendecomposition on **C**_*t*_ to obtain eigenvalues and eigenvectors

5:  Select top *b* eigenvectors from **C**_*t*_

6:  Concatenate selected eigenvectors from each subgroup

7: **end for**

8:  Apply mRMR feature selection on the concatenated eigenvectors (Algorithm 2)

9:  **return** Reduced data **Z**_*Q*_ with dimensions *X* × *Y* × *Q*

**Algorithm 2** mRMR Feature Selection

**Require:** Concatenated eigenvectors **Z** from Seg-PCA

1: Calculate mutual information between each feature and class labels

2: Calculate redundancy and relevance scores for each feature

3: Rank features based on mRMR score (relevance—redundancy)

4: Select top *b* features with highest mRMR scores

5: **return** Selected features

### 3.4 Neighbourhood extraction process

To execute our 3D-2D CNN model, the HSI data cube is divided into small overlapping 3D patches. These patches, denoted as *P*_*Q*_ = *s* × *s* × *Q*, are created from the Seg-PCA-mRMR reduced data cube **Z**_*Q*_ and are centered at spatial location (*i*, *j*). Each patch covers a spatial extent of *s* × *s* and contains all *Q* PCs representing the selected spectral features. The total number of generated 3D patches, denoted as *n*, from **Z**_*Q*_ is determined by (*X* − *s* + 1) × (*Y* − *s* + 1), where *X* and *Y* represent the spatial dimensions of the data cube. As a result, the 3D patch located at (*i*, *j*), denoted as *PQ*, *i*, *j*, includes the width from *i* − (*s* − 1)/2 to *i* + (*s* − 1)/2, height from *j* − (*s* − 1)/2 to *j* + (*s* − 1)/2, and contains *Q* selected features of the Seg-PCA-mRMR reduced data cube **Z**_*Q*_.

### 3.5 Spectral-spatial feature extraction with 3D-2D CNN

In our proposed spectral-spatial 3D-2D CNN architecture, we utilize three 3D convolution layers, one 2D convolution layer, and two fully connected layers. The 3D convolution operation involves convolving a 3D kernel with the input data to generate feature maps in the convolution layer. This process captures the spectral information present in multiple contiguous bands of the input layer. In the 3D convolution equation, the activation value at a spatial position (*i*, *j*, *k*) in the *f*-th feature map of the *l*-th layer, denoted as *x*^*i*, *j*, *k*^*l*, *f*, is computed by applying the activation function *ϕ* to the sum of the bias parameter *bl*, *f* and the weighted sum of the input values from the previous layer using the kernel weights *w*_*l*,*f*, *α*_. The kernel has a width of 2*ξ* + 1, a height of 2*δ* + 1, and a depth of 2*η* + 1 along with the spectral dimension, where *ξ*, *δ*, and *η* determine the kernel size. The weight parameter *w*_*l*,*f*, *α*_ represents the weight value for the *f*-th feature map of the *l*-th layer and the *α*-th spectral band. The computation of the activation function is carried out as follows:

$$\begin{array}{c} x_{l,f}^{i,j,k}=\phi \left(b_{l,f}+\sum_{\alpha =1}^{\lambda_{l-1}}\;\sum_{\beta =-\eta }^{\eta }\;\sum_{\gamma =-\xi }^{\xi }\;\sum_{\sigma =-\delta }^{\delta }w_{l,f,\alpha }^{\sigma ,\gamma ,\beta }*x_{l-1,\alpha }^{i+\sigma ,j+\gamma ,k+\beta }\right) \end{array}$$ (4)

In the case of 2D-CNN, the input data is convolved with 2D kernels. The convolution operation involves computing the sum of the dot product between the input data and the kernel, which is strived over the input data to cover the full spatial dimension. The resulting convolved features are then passed through the activation function to introduce nonlinearity in the model. In the equation for 2D convolution, the activation value at spatial position (*i*, *j*) in the *f*-th feature map of the *l*-th layer, denoted as *x*^*i*, *j*^*l*, *f*, is computed by applying the activation function *ϕ* to the sum of the bias parameter *bl*, *f* and the weighted sum of the input values from the previous layer using the kernel weights *w*_*l*,*f*, *α*_ as follows:

$$\begin{array}{c} x_{l,f}^{i,j}=\phi \left(b_{l,f}+\sum_{\alpha =1}^{\lambda_{l-1}}\;\sum_{\gamma =-\xi }^{\xi }\sum_{\sigma =-\delta }^{\delta }w_{l,f,\alpha }^{\sigma ,\gamma }*x_{l-1,\alpha }^{i+\sigma ,j+\gamma ,}\right) \end{array}$$ (5)

The kernel has a width of 2*ξ* + 1 and a height of 2*δ* + 1, and it does not include the spectral dimension as in the 3D convolution equation.

**Algorithm 3** 3D-2D CNN for Spectral-spatial Feature Extraction

**Require:** Reduced data cube **Z**_*Q*_ with dimensions *X* × *Y* × *Q*

1: Divide **Z**_*Q*_ into overlapping 3D patches *P*_*Q*_ of size *s* × *s* × *Q*

2: Initialize CNN architecture with specified parameters

3: Perform 3D convolution with kernel size 3 × 3 × 5 and 8 kernels

4: Perform 3D convolution with kernel size 3 × 3 × 3 and 16 kernels

5: Perform 3D convolution with kernel size 3 × 3 × 3 and 32 kernels

6: Perform 2D convolution with kernel size 3 × 3 and 64 kernels

7: Apply ReLU activation function after each convolution operation

8: Flatten the output tensor to prepare for fully connected layers

9: Add two fully connected layers with ReLU activation functions.

10: Output softmax probabilities for classification

11: **return** Classification accuracy

The 3D-2D CNN algorithm (Algorithm 3) is utilized in the proposed spectral-spatial feature extraction process, where it plays a crucial role in extracting discriminative features from the reduced data cube **Z**_*Q*_. These features are then utilized for subsequent classification tasks.

The parameters of the CNN, including bias and kernel weights, are trained using supervised approaches with gradient descent optimization. Conventional 2D CNNs operate on spatial dimensions only, limiting their ability to capture spectral information. On the other hand, 3D CNNs can extract both spatial and spectral features simultaneously, but at the cost of higher computational complexity. To leverage the feature learning capabilities of both 3D and 2D CNNs, our proposed spectral-spatial model combines them to extract different features and then fuses them for a more discriminative representation. The flattening layer is applied to preserve spatial information while reducing spectral dimensionality.

### 3.6 The model’s architecture and parameter details

The model’s architecture and parameter details are summarized in Table 1. The proposed model consists of convolutional layers with different kernel sizes and numbers of kernels, contributing to the extraction of different spatial and spectral features from the HSI data and enhancing the model’s discriminative power. The first 3D CNN has a kernel size of 3 × 3 × 5 and is composed of 8 kernels. The second 3D CNN has a kernel size of 3 × 3 × 3 and contains 16 kernels. The third 3D CNN has the same kernel size of 3 × 3 × 3 but contains 32 kernels. Finally, the 2D CNN has a kernel size of 3 × 3 and includes 64 kernels. The total trainable weight parameters for the proposed model is 4,824,816 for the Indian Pines (IP) dataset when *Q* = 10. These weights are randomly initialized and trained using the back-propagation algorithm with the Adam optimizer. The network was trained with a learning rate of 0.001, a decay rate of 0.000001, and mini-batch sizes of 256 over 100 epochs, without using batch normalization or data augmentation. The ReLU activation function is applied throughout the network, except in the output layer, as it efficiently captures non-linear relationships and aids in learning complex data representations. The output layer employs softmax activation for multi-class classification, ensuring the predicted probabilities for all classes sum to 1, which allows for accurate predictions.

**Table 1:** Layer-wise parameter details for the proposed model architecture with 25 × 25 window size based on IP dataset when *Q* = 10.

Layer Type	Output Shape	Number of Parameter
input (InputLayer)	25, 25, 10, 1	0
conv3d-1 (Conv3D)	23, 23, 6, 8	368
conv3d-2 (Conv3D)	21, 21, 4, 16	3472
conv3d-3 (Conv3D)	19, 19, 2, 32	13856
reshape (Reshape)	19, 19, 64	0
conv2d (Conv2D)	17, 17, 64	36928
flatten (Flatten)	18496	0
dense-1 (Dense)	256	4735232
dropout-1 (Dropout)	256	0
dense-2 (Dense)	128	32896
dropout-2 (Dropout)	128	0
output (Output)	16	2064
Total trainable parameters		4824816

## 4 Result and discussion

### 4.1 Experiment design and parameter setup

We divided the labeled samples into training and test subsets using different ratios for each dataset. The ratios used were 5%-95%, 10%-90%, 15%-85%, 20%-80% and 25%-75%. For example, in the case of a 25%-75% ratio, 25% of the data were randomly assigned to the training group, and the remaining 75% was assigned to the testing group. During the CNN training process, 90% of the training samples were used to determine the weights and biases of each neuron, and the remaining 10% were utilized to determine overfitting and inform network design choices.

In our proposed model, we first apply Seg-PCA-mRMR for spectral feature reduction by segmenting the HSI datasets into three segments based on average correlation analysis in the segmented subgroups (Table 2). After Seg-PCA, we scale the data using the min-max scaler from the Python sklearn preprocessing library.

**Table 2:** Details of datasets segmentation for Seg-PCA.

*L* No.	Parameter	IP	SA	PU
1	Range of bands	1-32	1-36	1-36
	Number of bands	32	36	36
2	Range of bands	33-72	37-106	37-72
	Number of bands	40	70	36
3	Range of bands	73-200	107-176	73-103
	Number of bands	128	70	31

*L* No. denote the segment number.

We extracted 3D patches from the input volume with a spatial dimension of 25 × 25 and *Q* spectral features to ensure a fair comparison, where *Q* represents the number of input spectral features. In our experiments, we applied Seg-PCA-mRMR for spectral feature reduction and selected the top-ranked 10 features based on mRMR. The order of the selected features for different datasets is shown in Table 3. For the model training, we set the learning rate to 0.001, the decay rate to 0.000001, the batch size to 256, the window size to 25 × 25 and epochs to 100, based on the classification performance. The model were trained with Adam optimizer and categorical cross-entropy loss function.

**Table 3:** Order of selected features by mRMR for different dataset.

Dataset	Order of selected features
IP	11, 12, 13, 15, 14, 1, 3, 2, 8, 6
SA	1, 4, 2, 3, 5, 10, 13, 11, 12, 8
PU	9, 8, 7, 6, 10, 14, 1, 4, 11, 13

To assess classification performance, we employed four standard quantitative metrics: Overall Accuracy (OA), Average Accuracy (AA), Kappa Coefficient, and F1 score. OA represents the proportion of correctly classified samples out of the total test samples, while AA calculates the mean accuracy across all classes. The Kappa Coefficient measures agreement between predicted and actual classifications, and the F1 score, as the harmonic mean of precision and recall, provides a balanced indication of a model’s reliability by giving equal weight to both metrics. Additionally, we monitored the training loss and training accuracy during the training process. Finally, we produced classification maps for further visualization of the results and to validate the effectiveness of the proposed method.

### 4.2 Classification results and analysis

The classification results were evaluated using three metrics: OA, AA, and Cohen’s Kappa, as described in the experimental design. The accuracies were measured in percentages for both the proposed method and state-of-the-art methods, including SVM on original data, Segmented PCA + SVM, 2D-CNN, Fast 3D-CNN, SpectralNet, HybridSN, Hybrid-2DNet, TP-Net, S2PNet, and DS2FE. These state-of-the-art experiments are described as follows:

- **2D-CNN** [25]: Applies randomized PCA for dimensionality reduction. It consists of two convolutional layers with filter counts of 30 and 90, respectively, followed by two fully connected layers with dropout rates of 25% and 50%, and an output layer.
- **Fast 3D-CNN** [41]: Utilizes incremental PCA for dimensionality reduction. The network comprises four convolutional layers with increasing filter counts and three fully connected layers.
- **SpectralNet** [39]: Applies factor analysis for dimensionality reduction and then executes a three-level Wavelet CNN decomposition.
- **HybridSN** [38]: Employs PCA for dimensionality reduction. It consists of three 3D convolution layers, one 2D convolution layer, and three fully connected layers.
- **Hybrid-2DNet** [43]: Utilizes factor analysis and mRMR-based feature selection followed by 2D-wavelet CNN in a four-level decomposition for reducing spectral and spatial dimensionalities. Additionally, batch normalization is applied.
- **TP-Net** [44]: Proposes a triple-path CNN with an attention mechanism to capture joint features.
- **S2PNet** [46]: An interactive learning approach used a multi-stage spectral purification module to reduce noise and spectral heterogeneity, while a global-local mutual guide module improved spatial feature discrimination.
- **DS2FE** [45]: Employs a vision-based transformer module that includes a multiscale feature extractor to capture joint spectral-spatial low-level and shallow features. For high-level semantic feature extraction, it uses a regional attention mechanism combined with a spatially gated module to enhance feature discrimination.

Table 4 displays the classification outcomes for ten spectral features across five different training-testing separations. The OA of the best-performing methods under various conditions is denoted in bold. The results indicate that our proposed method consistently outperforms other approaches in most scenarios.

**Table 4:** Classification results for the proposed and state-of-the-art methods.

Method	Training Samples	IP				SA				PU			
		OA	AA	Kappa	F1	OA	AA	Kappa	F1	OA	AA	Kappa	F1
Original data +SVM [9]	5%	45.17	25.25	33.45	38.54	69.98	65.22	66.10	68.26	62.34	38.17	45.10	54.35
	10%	46.11	26.17	34.72	39.61	70.93	67.13	67.22	69.24	62.49	38.43	45.27	55.12
	15%	46.43	26.34	35.21	40.08	71.47	68.07	67.81	69.86	62.63	38.46	45.52	55.31
	20%	46.44	26.89	35.41	40.27	71.74	68.46	68.13	70.16	62.62	38.53	45.56	55.35
	25%	46.81	27.30	35.95	40.71	71.93	68.66	68.34	70.37	62.80	39.16	45.81	55.59
Seg-PCA+SVM [15, 48, 50]	5%	64.12	53.06	58.51	61.32	91.58	94.96	90.69	91.10	86.20	75.29	81.18	83.66
	10%	70.57	59.80	65.92	68.23	92.16	95.56	91.26	91.67	88.53	78.52	84.49	85.63
	15%	73.64	63.52	69.53	72.15	92.55	95.59	91.68	92.09	89.81	81.48	86.27	87.45
	20%	75.55	67.36	71.78	74.36	92.71	96.13	91.87	92.21	90.61	83.82	87.39	88.56
	25%	76.80	69.52	73.26	75.87	92.95	96.31	92.12	92.46	91.44	85.49	88.52	90.69
2D-CNN [25]	5%	64.77	65.55	60.14	62.46	97.06	98.83	96.73	96.90	95.09	94.19	93.52	94.31
	10%	78.53	83.88	75.73	77.13	97.84	99.20	97.60	97.72	97.74	97.27	97.02	97.38
	15%	85.08	91.61	83.11	84.10	98.26	99.36	98.06	98.16	98.97	98.59	98.63	98.80
	20%	87.04	93.60	85.35	86.20	98.37	99.44	98.19	98.28	99.38	99.07	99.18	99.28
	25%	89.34	94.33	87.92	88.63	99.40	99.77	99.33	99.37	99.60	99.37	99.48	99.54
Fast 3D-CNN [41]	5%	55.18	36.31	46.93	51.06	93.32	96.24	92.56	92.94	89.79	82.31	86.34	88.07
	10%	70.58	50.96	65.98	68.28	96.75	98.37	96.38	96.57	95.18	92.32	93.60	94.39
	15%	62.02	45.40	55.68	58.85	99.54	99.82	99.48	99.51	97.57	96.10	96.77	97.17
	20%	80.83	66.18	77.95	79.39	99.96	99.98	99.96	99.96	99.15	98.57	98.88	99.02
	25%	85.49	79.03	83.42	84.46	99.83	99.77	99.81	99.82	99.01	98.18	98.69	98.85
SpectralNet [39]	5%	31.20	16.74	22.46	26.83	99.48	99.75	99.43	99.46	97.91	96.34	97.24	97.59
	10%	94.03	75.96	93.20	93.62	99.73	99.83	99.70	99.72	98.99	98.16	98.67	98.82
	15%	97.65	86.10	97.32	97.49	99.97	99.97	**99.97**	99.95	99.62	99.40	99.49	99.56
	20%	98.51	98.34	98.30	98.42	99.98	99.98	99.97	99.98	99.70	99.52	99.60	99.67
	25%	94.56	87.33	87.53	91.12	99.95	99.96	99.94	99.95	99.77	99.82	**99.71**	99.74
HybridSN [38]	5%	91.60	85.19	**91.41**	91.51	99.75	99.74	99.69	99.72	99.51	**99.07**	99.35	99.43
	10%	97.59	**97.07**	97.25	97.42	99.92	99.91	99.92	99.92	99.48	98.97	**99.48**	99.48
	15%	98.88	99.22	98.73	98.81	99.95	99.86	99.95	99.95	99.61	99.50	99.49	99.55
	20%	98.94	98.27	98.79	98.87	99.98	99.98	99.98	99.98	99.75	99.70	99.73	99.74
	25%	99.49	99.31	99.42	99.46	99.97	99.95	99.97	99.97	98.99	98.99	98.99	98.99
Hybrid-2DNet [43]	5%	35.41	19.01	23.31	29.37	99.76	99.86	99.75	99.76	96.33	95.51	95.11	95.72
	10%	94.04	76.10	93.21	93.63	99.94	99.94	99.94	99.94	98.91	97.91	98.43	98.67
	15%	97.67	97.65	97.29	97.48	**99.97**	99.97	**99.97**	99.97	99.64	99.41	99.51	99.58
	20%	98.58	88.07	98.38	98.49	99.99	99.98	99.98	99.99	99.72	99.47	99.57	99.65
	25%	97.79	98.01	98.43	98.11	99.97	99.96	99.97	99.97	99.78	99.62	99.68	99.73
TP-Net [44]	5%	37.11	23.13	27.42	32.29	99.61	99.65	99.60	99.61	**99.53**	99.05	**99.38**	99.46
	10%	86.51	79.58	81.37	83.95	99.93	99.93	99.93	99.93	**99.55**	98.97	99.45	99.50
	15%	93.56	89.77	91.84	92.71	99.95	99.91	99.95	99.95	99.67	99.65	99.61	99.64
	20%	98.71	98.36	98.44	98.57	99.98	99.96	99.97	99.98	99.85	99.34	99.23	99.54
	25%	97.16	93.94	96.19	96.66	99.96	99.96	99.95	99.96	99.79	99.82	99.70	99.75
S2PNet [46]	5%	92.38	**89.07**	90.48	91.63	99.78	99.74	99.57	99.69	98.45	98.38	98.17	98.31
	10%	97.77	94.71	95.84	96.24	99.86	99.82	99.67	99.76	98.71	98.69	98.49	98.64
	15%	98.59	96.02	96.45	97.16	99.88	99.84	99.66	99.79	99.32	99.28	98.71	98.85
	20%	98.92	98.91	97.62	97.98	99.95	99.96	99.82	99.85	99.85	99.84	99.68	99.70
	25%	99.48	99.47	99.07	99.21	99.92	99.92	99.80	99.83	99.21	99.22	99.06	99.17
DS2FE [45]	5%	**92.39**	89.02	90.41	**91.72**	99.81	99.80	99.63	99.72	98.40	98.33	98.15	98.28
	10%	97.85	94.78	95.95	96.36	99.89	99.88	99.71	99.84	98.82	98.80	98.61	98.73
	15%	98.63	96.14	96.56	97.22	99.94	99.93	99.81	99.87	99.43	99.39	98.85	98.94
	20%	**98.97**	98.97	97.68	98.03	99.98	99.97	99.90	99.92	99.91	99.85	99.67	99.71
	25%	99.45	99.46	99.08	99.20	99.94	99.94	99.88	99.90	99.37	99.31	98.97	99.15
Proposed Method	5%	92.36	88.76	90.97	91.69	**99.78**	**99.85**	**99.76**	99.77	99.19	98.41	98.28	99.74
	10%	**97.85**	96.52	**97.76**	97.82	**99.96**	**99.96**	**99.96**	99.96	99.53	**98.98**	99.35	99.44
	15%	**98.96**	**98.22**	**98.86**	98.92	**99.97**	**99.98**	99.96	99.97	**99.76**	**99.71**	**99.68**	99.72
	20%	**99.06**	98.16	**98.93**	99.02	**100**	**99.99**	**100**	100	**99.94**	**99.93**	**99.92**	99.93
	25%	**99.61**	**99.65**	**99.55**	99.59	**99.98**	**99.99**	**99.98**	99.98	**99.85**	**99.89**	99.14	99.50

Among the three datasets, the PU dataset exhibits the lowest number of categories, comprising only nine classes, thereby making it comparatively easier to classify than the other datasets. In contrast, the SA dataset features a larger spatial size and the maximum number of spectral bands, providing more discriminative information for classification. Consequently, the listed methods generally demonstrate higher classification performance on the SA dataset. The IP dataset, although having a relatively small spatial size, encompasses 16 categories, presenting a challenge for accurate classification and resulting in lower accuracy compared to the other datasets. The traditional SVM model applied directly to the original data yields inadequate classification performance. However, upon preprocessing the data using Seg-PCA and integrating it with SVM, there is a notable enhancement in classification accuracy.

The 2D-CNN and Fast 3D-CNN methods demonstrate lower classification performances, possibly attributable to their limited utilization of spectral information. Conversely, SpectralNet achieves moderate classification performances by incorporating both spectral and spatial features. HybridSN, which combines several 3D CNNs with a 2D CNN, is a simple and effective approach, yielding discriminative features. The Hybrid-2DNet method demonstrates a notable performance improvement over SpectralNet, highlighting the crucial role of feature selection. However, it does not consistently surpass HybridSN across all scenarios. In contrast, TP-Net consistently outperforms both SpectralNet and HybridSN in most cases, indicating its superior performance. S2PNet performs comparably to TP-Net, while DS2FE surpasses TP-Net in most instances due to its ability to handle joint spectral-spatial low-level and shallow features, along with high-level semantic feature extraction using a regional attention mechanism.

Indeed, the proposed approach capitalizes on the combined potential of spatial and spectral features within HSI data. Through the extraction of distinct image features from segmented subgroups’ PCs and their subsequent fusion, the network acquires a rich array of representative and differentiating features. This comprehensive feature set contributes to an enhanced classification performance overall. By leveraging both spatial and spectral information, and harnessing the benefits of feature fusion, the proposed method achieves an advanced level of classification accuracy.

Additionally, the class-wise classification accuracies in percentages are shown in Tables 5–7 with top 10 selected features and 20% training samples for all three experimented datasets. The results in Tables 4–7 demonstrate that the proposed methods offer the best OA of 99.61%, 100%, and 99.94% for IP, SA, and PU datasets, respectively. In comparison, the DS2FE, S2PNet, TP-Net, Hybrid-2DNet, HybridSN, and SpectralNet models achieve their optimal results with OAs of 98.97%, 98.92%, 98.71%, 98.58%, 99.49% and 98.51% for IP, 99.98%, 99.95%, 99.98%, 99.99%, 99.98% and 99.98% for SA, and 99.91%, 99.85%, 99.85%, 99.78%, 99.75% and 99.70% for PU datasets under the same spectral features conditions. These results indicate that the proposed method significantly improves the classification accuracy compared to the state-of-the-art models under the same spectral features and training sample conditions.

**Table 5:** Class-wise classification accuracies (%) of IP with 10 spectral features and 20% training samples.

Classes	Original data+SVM	Seg-PCA+SVM	2D-CNN	Fast 3D-CNN	SpectralNet	HybridSN	Hybrid-2DNET	TP-Net	S2PNet	DS2FE	Proposed Method
Alfalfa	0.00	42.56	92.50	99.74	99.83	92.50	80.43	**100**	**100**	**100**	**100**
Corn-notill	43.25	65.37	85.71	65.80	**99.73**	99.36	98.50	96.77	96.77	97.50	98.24
Corn-mintill	14.58	70.62	83.44	79.38	97.21	98.95	98.36	97.92	98.36	98.95	**99.25**
Corn	0.00	57.84	93.75	85.71	99.48	**100**	97.88	**100**	97.88	98.50	98.96
Grass-pasture	8.12	88.79	95.21	84.99	**99.85**	99.74	98.71	**100**	98.71	99.74	99.23
Grass-trees	47.37	87.20	87.81	79.67	99.83	**100**	99.66	94.19	99.81	**100**	98.81
Grass-pasture-mowed	0.00	71.00	95.65	**100**	**100**	**100**	**100**	**100**	**100**	**100**	**100**
Hay-windrowed	31.12	90.87	90.09	90.28	99.74	99.74	99.22	99.71	99.74	99.74	**99.78**
Oats	0.00	31.00	76.19	**100**	0.00	**100**	**100**	**100**	**100**	**100**	**100**
Soybean-notill	49.50	69.50	79.31	77.51	98.73	97.74	99.35	99.59	99.75	99.59	**100**
Soybean-mintill	39.37	68.75	89.53	81.38	97.61	98.25	98.49	**99.20**	98.49	98.25	98.84
Soybean-clean	0.00	67.87	80.28	69.52	98.52	98.73	**99.57**	94.10	98.52	94.10	98.92
Wheat	0.00	91.12	95.35	93.37	**100**	**100**	**100**	**100**	**100**	**100**	**100**
Woods	72.25	94.33	97.82	98.56	99.61	99.70	97.97	97.07	99.70	98.56	**100**
Buildings-GTD	0.00	70.57	68.47	85.04	98.72	**100**	99.36	97.39	97.39	98.72	**100**
Stone-Steel-Towers	**100**	98.08	91.36	88.89	97.10	88.75	93.59	**100**	93.59	97.10	86.75
**OA**	46.44	75.55	87.04	80.83	98.51	98.94	98.58	98.71	98.92	98.97	**99.06**
**AA**	26.89	67.36	93.60	66.18	98.34	98.27	88.07	98.36	98.91	**98.97**	98.16
**Kappa**	35.41	71.78	85.35	77.95	98.30	98.79	98.38	98.44	97.62	97.68	**98.93**

**Table 6:** Class-wise classification accuracies (%) of SA with 10 spectral features and 20% training samples.

Classes	Original data+SVM	SegPCA+SVM	2D-CNN	Fast 3D-CNN	SpectralNet	HybridSN	Hybrid-2DNET	TP-Net	S2PNet	DS2FE	Proposed Method
Brocoli-green-weeds-1	91.71	**100**	**100**	**100**	**100**	**100**	**100**	**100**	**100**	**100**	**100**
Brocoli-green-weeds-2	90.28	**100**	99.90	**100**	**100**	99.97	**100**	**100**	99.75	**100**	99.98
Fallow	53.23	97.07	**100**	**100**	**100**	**100**	**100**	**100**	**100**	**100**	**100**
Fallow-rough-plow	61.55	98.74	99.64	99.91	98.95	99.07	**100**	99.91	99.07	99.91	**100**
Fallow-smooth	60.21	99.14	99.78	99.84	**100**	**100**	**100**	99.81	**100**	**100**	**100**
Stubble	96.68	**100**	99.97	**100**	99.97	**100**	**100**	99.94	99.94	**100**	**100**
Celery	90.16	**100**	99.93	99.87	**100**	**100**	**100**	**100**	**100**	**100**	**100**
Grapes-untrained	57.56	78.52	98.86	99.91	98.74	99.79	99.78	**100**	**100**	99.91	**100**
Soil-vinyard-develop	82.34	99.58	**100**	**100**	**100**	**100**	**100**	**100**	**100**	**100**	**100**
Corn-senesced-GW	60.10	96.85	**100**	99.96	**100**	**100**	**100**	**100**	**100**	**100**	**100**
Lettuce-romaine-4wk	0.00	98.63	99.78	99.86	99.94	99.98	**100**	**100**	99.94	**100**	**100**
Lettuce-romaine-5wk	83.03	98.17	**100**	**100**	**100**	**100**	**100**	**100**	**100**	**100**	**100**
Lettuce-romaine-6wk	68.08	98.67	**100**	**100**	**100**	**100**	**100**	99.86	99.67	**100**	**100**
Lettuce-romaine-7wk	66.42	98.14	**100**	**100**	**100**	99.83	**100**	**100**	**100**	**100**	**100**
Vinyard-untrained	54.53	82.50	90.50	99.91	99.16	**100**	99.67	99.97	**100**	**100**	**100**
Vinyard-vertical-trellis	82.57	**100**	**100**	**100**	**100**	**100**	**100**	**100**	**100**	**100**	**100**
**OA**	71.74	92.71	98.37	99.96	99.98	99.98	99.99	99.98	99.95	99.98	**100**
**AA**	68.46	96.13	99.44	99.98	99.98	99.98	99.98	99.96	99.96	99.97	**99.99**
**Kappa**	68.13	91.87	98.19	99.96	99.97	99.98	99.98	99.97	99.82	99.90	**100**

**Table 7:** Class-wise classification accuracies (%) of PU with 10 spectral features and 20% training samples.

Classes	Original data+SVM	SegPCA+SVM	2D-CNN	Fast 3D-CNN	SpectralNet	HybridSN	Hybrid-2DNET	TP-Net	S2PNet	DS2FE	Proposed Method
Asphalt	53.96	84.76	99.70	99.05	99.92	99.74	99.42	99.96	99.96	99.96	**99.98**
Meadows	68.47	92.57	99.92	99.67	99.75	99.55	99.96	99.79	99.96	99.96	**99.99**
Gravel	39.54	83.45	97.54	98.98	98.78	**100**	99.16	99.35	99.16	99.35	**100**
Trees	0.00	96.10	98.65	98.63	99.58	99.59	98.94	98.82	98.82	98.94	**100**
Painted MS	94.67	100	100	99.72	**100**	**100**	**100**	**100**	**100**	**100**	**100**
Bare Soil	40.78	92.84	99.80	99.73	**100**	99.98	99.88	**100**	**100**	**100**	**100**
Bitumen	0.00	93.07	98.70	98.02	99.18	99.61	99.81	**100**	**100**	**100**	99.81
Self-BB	47.18	80.58	97.43	97.10	98.82	98.90	99.32	98.81	98.90	98.82	**99.49**
Shadows	0.00	**100**	99.47	97.75	**100**	99.74	99.73	98.78	99.73	**100**	**100**
**OA**	62.62	90.61	99.38	99.15	99.70	99.75	99.72	99.85	99.85	99.91	**99.94**
**AA**	38.53	83.82	99.07	98.57	99.52	99.70	99.47	99.34	99.84	99.85	**99.93**
**Kappa**	45.56	87.39	99.18	98.88	99.60	99.73	99.57	99.23	99.68	99.67	**99.92**

**Fig 2:**
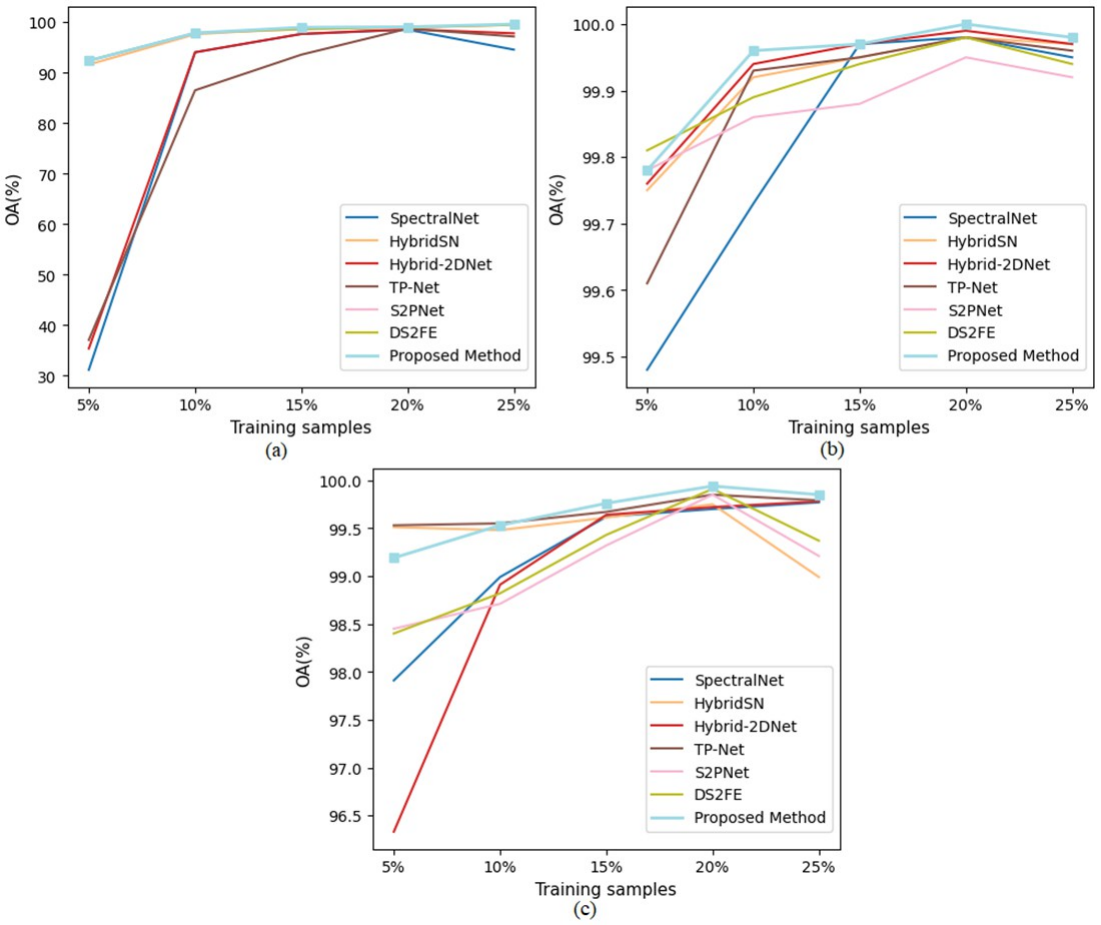
Overall accuracy comparison of state-of-the-art methods with the proposed method as the number of training samples increases for (a) IP, (b) SA, and (c) PU datasets.

Fig 2 shows the overall accuracy performances of state-of-the-art methods with the proposed method as the number of training samples increases for different datasets. It is evident from the results in the tables and Fig 2 that the proposed methods consistently exhibit significantly better performance compared to SVM on original data and Seg-PCA + SVM methods for all three datasets. Similarly, our proposed methods clearly outperform the 2D-CNN, Fast 3D-CNN, and SpectralNet methods across all three datasets. Furthermore, in comparison to HybridSN, Hybrid-2DNet, DS2FE, S2PNet, and TP-Net the proposed methods demonstrate notable improvements. Overall, the results consistently indicate that the proposed methods outperform or surpass the performance of several benchmark methods across all three datasets. Moreover, as the number of training samples increases, all models show improved performance, except for when using 25% training samples, which sometimes exhibits lower accuracy compared to the performance with 20% training samples. This observation suggests that a larger training dataset allows the models to learn more representative and discriminative features, thus enhancing classification accuracy.

**Fig 3:**
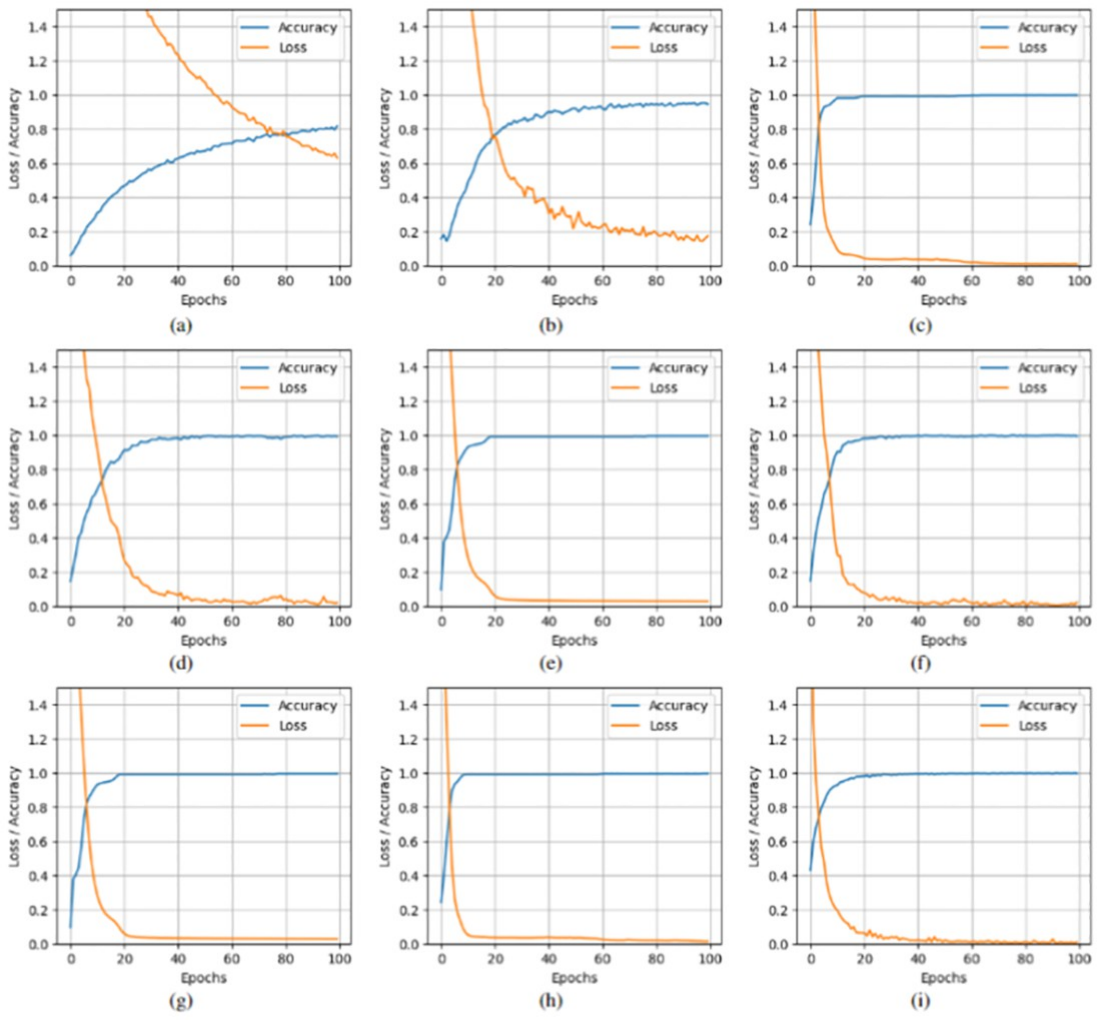
IP data, (a)-(i) Accuracy, and Loss convergence versus the number of epochs over top 10 selected features and 20% training samples, (a) 2D CNN, (b) Fast 3D-CNN, (c) SpectralNet, (d) HybridSN, (e) Hybrid-2DNet, (f) TP-Net, (g) S2PNet, (h) DS2FE and (i) Proposed Method.

Fig 3 illustrates the convergence of accuracy and loss for the proposed and state-of-the-art models on the IP dataset, using 20% of training samples over 100 epochs. Our method achieves convergence around the 18th epoch, demonstrating a relatively fast convergence rate compared to other models, except for S2PNet and DS2FE, which converge faster. This highlights the efficiency and effectiveness of our approach in capturing data patterns, resulting in improved classification performance.

### 4.3 Classification maps

**Fig 4:**
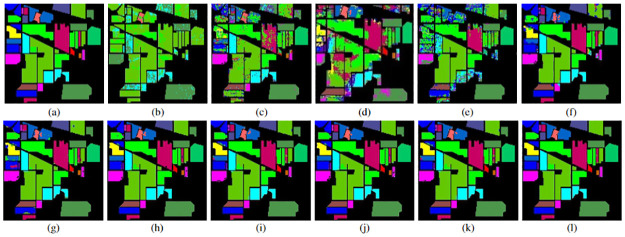
IP HSI (a) ground truth image, (b)-(h) classification maps for different SVM and CNN models with top 10 selected features and 20% training sample. (b) Original data + SVM, (c) Seg-PCA + SVM, (d) 2D CNN, (e) Fast 3D-CNN, (f) SpectralNet, (g) HybridSN, (h) Hybrid-2DNet, (i)TP-Net, (j) S2PNet, (k) DS2FE, and (l) Proposed Method.

**Fig 5:**
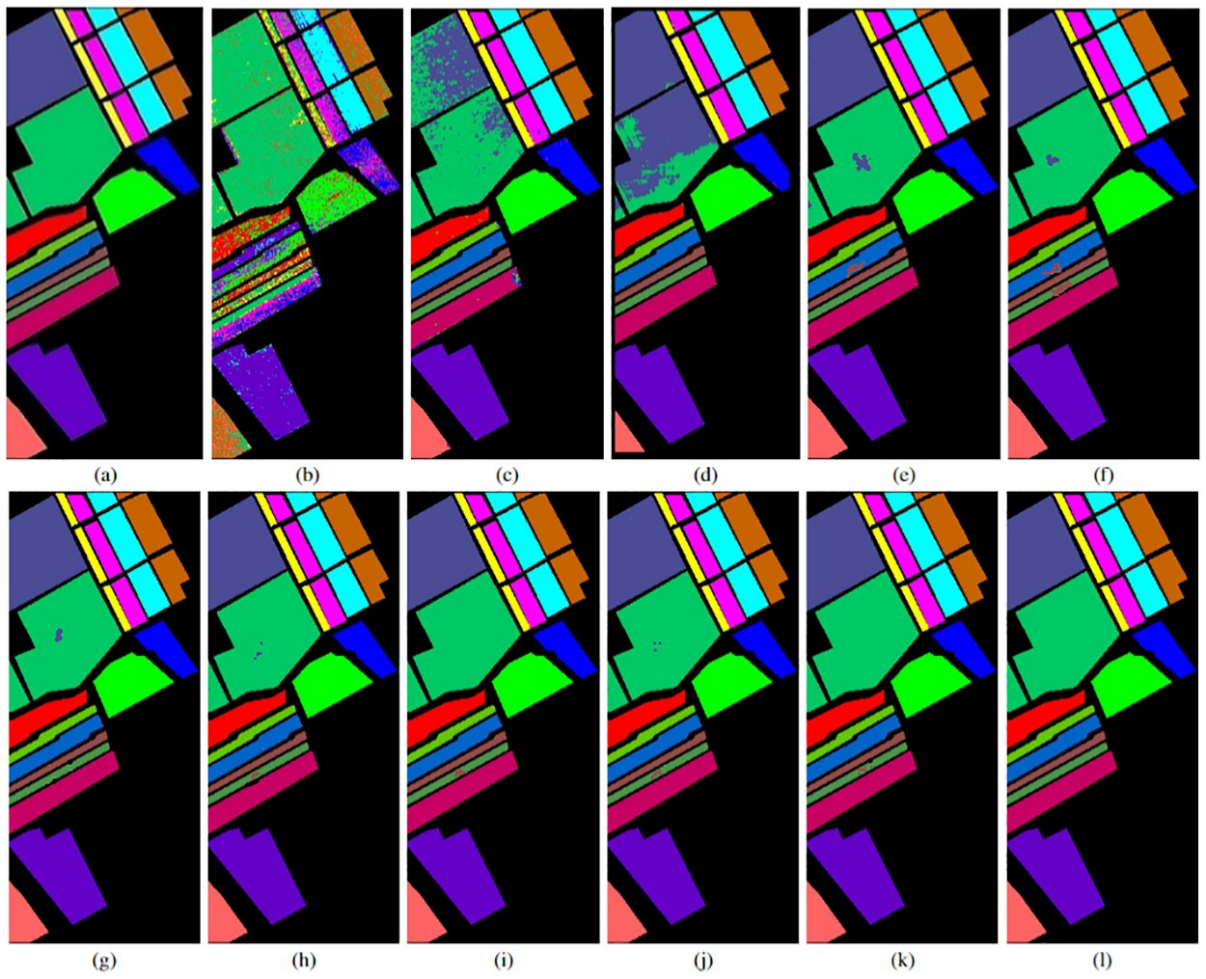
SA HSI (a) ground truth image, (b)-(h) classification maps for different SVM and CNN models with top 10 selected features and 20% training sample. (b) Original data + SVM, (c) Seg-PCA + SVM, (d) 2D CNN, (e) Fast 3D-CNN, (f) SpectralNet, (g) HybridSN, (h) Hybrid-2DNet, (i)TP-Net, (j) S2PNet, (k) DS2FE, and (l) Proposed Method.

**Fig 6:**
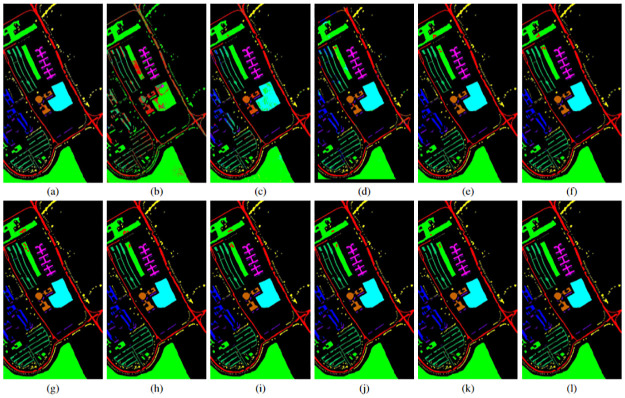
PU HSI (a) ground truth image, (b)-(h) classification maps for different SVM and CNN models with top 10 selected features and 20% training sample. (b) Original data + SVM, (c) Seg-PCA + SVM, (d) 2D CNN, (e) Fast 3D-CNN, (f) SpectralNet, (g) HybridSN, (h) Hybrid-2DNet, (i)TP-Net, (j) S2PNet, (k) DS2FE, and (l) Proposed Method.

In addition to the quantitative evaluation, a visual assessment of classification maps is performed to validate the effectiveness of the proposed method. Figs 4–6 showcase the classification maps obtained by different methods for various HSIs. These maps are generated using 20% of the training samples for both the proposed and the other studied methods. Upon visual inspection, it becomes evident that the proposed method consistently outperforms the other approaches across all datasets. The classification maps produced by the proposed method exhibit higher clarity and fewer misclassified pixels, demonstrating its superior performance in capturing the complex spatial and spectral information present in hyperspectral imagery. The visually compelling results further validate the efficacy of our proposed method and reinforce its potential for accurate HSI classification tasks.

### 4.4 Ablation analysis

A comprehensive ablation analysis was conducted using the novel integrated approach (Seg-PCA + mRMR + 3D-2D CNN) across five distinct subgroups. The first experiment evaluated dataset segmentation before PCA, comparing the performance of conventional PCA (PCA + 3D-2D CNN) with Seg-PCA (Seg-PCA + 3D-2D CNN), without mRMR-based feature selection. The second experiment assessed the impact of mRMR-based feature selection on both conventional PCA and Seg-PCA. Compared to group 1, the efficacy of mRMR-based feature selection is clearly demonstrated, showing its impact on improving the model’s performance. The third experiment compared the deep 3D-2D model’s performance with traditional machine learning (SVM). In the fourth, the individual performance of 2D and 3D CNNs was evaluated, and the fifth experiment examined the influence of the number of 3D CNN layers in the 3D-2D CNN model.

The ablation analysis results, detailed in Table 8, demonstrate that both Seg-PCA and mRMR feature selection significantly improved classification accuracy compared to classical PCA and the absence of feature selection. The findings also determine the optimal number of 3D CNN layers and the advantage of deep 3D-2D CNN over traditional machine learning. Notably, combining Seg-PCA and mRMR produced a synergistic effect, greatly enhancing overall classification performance, underscoring the importance of these techniques both individually and together in the integrated framework.

**Table 8:** Classification results for ablation analysis with 10 spectral features.

Ablation Group	Ablation Method	Training Samples	IP			SA			PU		
			OA	AA	Kappa	OA	AA	Kappa	OA	AA	Kappa
Group 1	PCA + 3D-2D CNN (Three 3D CNN)	10%	97.54	96.30	97.22	99.91	99.90	99.91	99.31	98.84	99.03
		20%	98.82	97.96	98.79	99.96	99.96	99.95	99.81	99.78	99.80
	Seg-PCA + 3D-2D CNN (Three 3D CNN)	10%	97.60	96.37	97.27	99.92	99.92	99.92	99.39	98.90	99.15
		20%	98.95	98.12	98.95	99.98	99.97	99.98	99.88	99.86	99.88
Group 2	PCA + mRMR + 3D-2D CNN (Three 3D CNN)	10%	97.61	96.33	97.26	99.93	99.92	99.93	99.38	98.92	99.16
		20%	98.96	98.11	98.95	99.98	99.98	99.98	99.89	99.85	99.88
	Seg-PCA + mRMR + 3D-2D CNN (Three 3D CNN)(Proposed)	10%	97.85	96.52	97.76	99.96	99.96	99.96	99.53	98.98	99.35
		20%	99.06	98.16	98.93	100	99.99	100	99.94	99.93	99.92
Group 3	Seg-PCA + SVM	10%	46.11	26.17	34.72	70.93	67.13	67.22	62.49	38.43	45.27
		20%	46.44	26.89	35.41	71.74	68.46	68.13	62.62	38.53	45.56
	Seg-PCA + 3D-2D CNN (Three 3D CNN)	10%	97.60	96.37	97.27	99.92	99.92	99.92	99.39	98.90	99.15
		20%	98.95	98.12	98.95	99.98	99.97	99.98	99.88	99.86	99.88
Group 4	Seg-PCA + mRMR + 2D CNN (No 3D CNN)	10%	92.94	86.17	91.93	99.82	99.83	99.80	99.43	99.37	99.38
		20%	97.68	97.61	97.35	99.83	99.84	99.82	99.73	99.70	99.71
	Seg-PCA + mRMR + 3D CNN (No 2D CNN, Three 3D CNN)	10%	95.71	88.81	95.10	99.78	99.78	99.77	99.45	99.36	99.39
		20%	98.61	98.46	98.41	99.79	99.84	99.77	99.78	99.68	99.72
Group 5	Seg-PCA + mRMR + 3D-2D CNN (One 3D CNN)	10%	96.25	89.54	95.72	99.82	99.81	99.77	99.41	98.85	99.04
		20%	98.43	97.48	98.21	99.96	99.96	99.95	99.89	99.84	99.87
	Seg-PCA + mRMR + 3D-2D CNN (Two 3D CNN)	10%	97.15	90.29	96.18	99.88	99.87	99.83	99.48	99.05	99.28
		20%	98.95	98.01	98.80	99.97	99.97	99.96	99.92	99.91	99.90
	Seg-PCA + mRMR + 3D-2D CNN (Three 3D CNN)(Proposed)	10%	97.85	96.52	97.76	99.96	99.96	99.96	99.53	98.98	99.35
		20%	99.06	98.16	98.93	100	99.99	100	99.94	99.93	99.92

### 4.5 Computation time analysis

All experiments were performed on Google Colaboratory using a setup consisting of 2 x vCPUs and a maximum of 25 (mostly 12) GB RAM. In order to ensure a fair and accurate comparison, we applied the same spatial dimension and spectral features extraction technique to obtain 3D patches from various input data. Throughout the experiments, we measured three types of computation times for both the different studies and our proposed method. These computation times are denoted as Pp for preprocessing, Tr for training, and Ts for testing. The results of these time measurements are presented in Table 9, with the time values given in seconds, minutes, and seconds, respectively. It is worth noting that our proposed model demonstrated higher computational efficiency compared to the fast 3D-CNN, SpectralNet, HybridSN, Hybrid-2DNet, TP-Net, DS2FE, and S2PNet, methods when considering the preprocessing, training, and testing times. However, fusion methods DS2FE, S2PNet, Hybrid-2DNet and TP-Net show better classification performances than SpectralNet, and HybridSN but face more computational complexity. Only 2D-CNN offers better computational efficiency than the proposed methods but gives lower classification accuracy.

**Table 9:** Different stages computation time, prepossessing (Pp) time in seconds (s), training (Tr) time in minutes (m), testing (Ts) in seconds (s), for the proposed and state-of-the-art methods.

Method	Training Samples	IP			SA			PU		
		Pp(s)	Tr(m)	Ts(s)	Pp(s)	Tr(m)	Ts(s)	Pp(s)	Tr(m)	Ts(s)
2D-CNN [25]	10%	8.1	3.8	5.4	23.5	5.9	8.2	25.3	4.6	6.8
	20%	8.1	7.3	4.8	23.5	10.2	7.6	25.3	8.9	5.7
Fast 3D-CNN [41]	10%	6.2	31.5	58.2	15.7	216.2	356.8	21.6	108.5	368.6
	20%	6.2	45.4	51.7	15.7	428.3	316.4	21.6	211.6	327.4
SpectralNet [39]	10%	25.8	23.1	19.6	75.1	121.8	103.3	54.6	73.1	61.8
	20%	25.8	43.8	17.4	75.1	226.1	91.8	54.6	135.4	56.7
HybridSN [38]	10%	6.8	16.2	32.7	28.2	103.8	201.4	31.1	51.4	103.7
	20%	6.8	30.1	29.1	28.2	194.7	179.0	31.1	96.2	92.2
Hybrid-2DNET [43]	10%	47.8	22.5	20.4	92.3	118.1	105.6	59.7	78.2	62.3
	20%	47.8	40.7	16.9	92.3	223.5	92.4	59.7	138.6	55.6
TP-NET [44]	10%	6.8	31.5	61.6	29.4	197.9	187.4	32.4	101.2	188.4
	20%	6.8	57.6	54.7	29.4	357.8	155.6	32.4	182.4	174.7
S2PNet [46]	10%	67.4	29.6	47.1	127.5	155.1	143.8	92.3	108.2	92.3
	20%	67.4	55.8	35.2	127.5	307.5	116.7	92.3	198.6	75.6
DS2FE [45]	10%	38.6	28.5	45.4	93.1	142.7	137.8	74.1	88.7	176.8
	20%	38.6	55.4	33.3	93.1	281.7	113.5	74.1	171.4	139.9
Proposed Method	10%	7.6	**9.8**	19.1	30.2	**65.3**	135.6	34.2	**37.5**	36.9
	20%	7.6	**13.4**	17.0	30.2	**122.5**	120.2	34.4	**68.3**	32.8

### 4.6 Discussion

The experimental results highlight the challenges of limited training samples and high dimensionality in HSI classification, common in real-world applications where acquiring labeled data is difficult and resource-intensive. Despite these challenges, the proposed method effectively overcomes them by combining segmented principal component analysis (Seg-PCA) for feature extraction with 3D-2D CNNs for joint spectral-spatial feature learning. This hybrid approach addresses both the scalability and robustness issues, showing improved performance compared to traditional CNN-based methods. In addressing the limitations of traditional approaches, the integration of Seg-PCA with mRMR-based feature selection enhances the model’s ability to handle high-dimensional HSI data by reducing redundant information and focusing on key features. The incorporation of both 3D and 2D CNN layers balances computational efficiency with feature extraction depth, allowing the model to adapt effectively to variations in dataset characteristics. As a result, the proposed method achieves better classification accuracy and generalization across different datasets, while also mitigating the computational complexity of deep 3D CNNs.

This research demonstrating the model’s architecture remains efficient and adaptable as data complexity increases. Additionally, the robustness of the proposed method is evident in its generalizability across three widely used HSI datasets, outperforming state-of-the-art methods such as SVM, 2D-CNN, Fast 3D-CNN, SpectralNet, HybridSN, Hybrid-2DNet, TP-Net, 2PNet, and DS2FE. The results in tables and figures as well as classification maps indicate that the combination of Seg-PCA and 3D-2D CNNs significantly contributes to overcoming the limitations posed by traditional CNNs, particularly in handling high-dimensional data with limited training samples. In respect of computation time the proposed model offer relatively higher computational efficiency compared to the state of the art.

While our proposed method demonstrates significant improvements in HSI classification, it does have some limitations that offer avenues for future research. Despite the integration of both 3D and 2D CNN layers to balance computational efficiency, the overall computational cost remains relatively high, particularly when applied to large-scale datasets. The complexity could limit the scalability of the model for such real-time applications where large-scale datasets involve. Although the model generalizes well across the evaluated datasets, further validation on additional, more diverse datasets is necessary to confirm its adaptability to varying environments and spectral conditions. However, expanding the application of the proposed model to diverse fields such as precision agriculture, environmental monitoring, and urban mapping could validate its robustness and scalability across various real-world scenarios. Additionally, the model could be tailored for specific domain requirements, further improving its performance in specialized tasks.

## 5 Conclusion and future work

Land cover object classification is a vital task in remote sensing and has significant implications for fields such as environmental monitoring, urban planning, and agriculture. Despite the advancements in HSI classification, achieving high accuracy remains challenging due to issues like high dimensionality and the limited availability of training samples. Traditional methods often focus on spectral information, which may overlook critical spatial features. To address these limitations, we introduced a novel HSI classification approach that enhances land cover object classification by integrating Seg-PCA with mRMR feature selection and a hybrid 3D-2D CNN architecture. Our proposed method effectively combines Seg-PCA for localized spectral feature extraction with mRMR for feature selection, followed by a 3D-2D CNN framework that captures both spectral and spatial features. This approach not only addresses the challenges of high dimensionality and limited training data but also significantly improves classification performance for land cover object classification tasks. The extensive experiments conducted on three benchmark HSI datasets demonstrate that our method outperforms existing state-of-the-art techniques in terms of OA, AA, and Cohen’s Kappa statistic, proving its effectiveness and robustness. Looking ahead, several avenues for future research could further enhance HSI classification techniques. One potential direction is the exploration of advanced feature fusion strategies that integrate additional data modalities, such as LiDAR or multispectral imagery, to improve classification accuracy and robustness. Another promising area is the development of more efficient network architectures and training algorithms that can handle even larger datasets and more complex classification tasks, potentially leveraging transfer learning or few-shot learning techniques to make better use of limited training data. Finally, our approach sets a new benchmark for land cover object classification in hyperspectral imagery and offers a foundation for future innovations in the field of hyperspectral remote sensing.
